# BOLD-Perfusion Coupling during Monocular and Binocular Stimulation

**DOI:** 10.1155/2008/628718

**Published:** 2008-03-02

**Authors:** Claudine Gauthier, Richard D. Hoge

**Affiliations:** ^1^Unité de Neuroimagerie Fonctionnelle, Institut Universitairy of Gériatrie de Montréal, Montreal, QC, Canada H3W 1W5; ^2^Department of Physiology, Faculty of Medicine, University of Montreal, Montreal, QC, Canada H3W 1W5; ^3^Institute of Biomedical Engineering, Faculty of Medicine, University of Montreal, Montreal, QC, Canada H3W 1W5

## Abstract

Previous studies have suggested that during selective activation of a subset of the zones comprising a columnar system in visual cortex, perfusion increases uniformly in all columns of the system, while increases in oxidative metabolism occur predominantly in the activated columns. This could lead to disproportionately large blood oxygenation level-dependent (BOLD) signal increases for a given flow increase during monocular (relative to binocular) stimulation, due to contributions from columns which undergo large increases in perfusion with little or no change in oxidative metabolism. In the present study, we sought to test this hypothesis by measuring BOLD-perfusion coupling ratios in spatially averaged signals over V1 during monocular and binocular visual stimulation. It was found that, although withholding input to one eye resulted in statistically significant decreases in BOLD and perfusion signals in primary visual cortex, the ratio between BOLD and perfusion increases did not change significantly. These results do not support a gross mismatch between spatial patterns of flow and metabolism response during monocular stimulation.

## 1. INTRODUCTION

Although blood oxygenation level-dependent (BOLD) functional MRI has assumed a role of great importance in systems neuroscience, our understanding of factors determining the amplitude and spatial extent of the BOLD effect under different conditions remains incomplete. Relevant parameters include baseline values and reactive capacity for cerebral perfusion, oxidative metabolism, and blood volume. An understanding of how these contribute to the BOLD response is important, since in general they may vary due to age or disease, and also depending on the nature of the neural system targeted by an applied stimulus. In particular, the exact nature and extent of the coupling between changes in oxidative metabolism and perfusion increases during neuronal activation is still the subject of debate. While recent studies have focused on quantification of responses during nonspecific activation of diffuse regions of sensory and motor cortex [1–4], this topic has also arisen in the context of highly localized responses in cortical columnar systems [5, 6]. In the present study, we sought to bridge the gap between these two regimes by looking at the effect of selective activation of only part of a small-scale cortical columnar system on the apparent BOLD response observed at a spatial resolution typical of studies used in human subjects.

Early optical imaging studies [7–9] suggested that although evoked changes in oxidative metabolism exhibit a high degree of spatial specificity, brain perfusion is regulated on a much coarser spatial scale. If this is true, then there might be profound implications for the BOLD MRI signal, especially when measured during manipulations such as monocular stimulation, which preferentially activates the set of ocular dominance columns projecting to the stimulated eye. In particular, one might expect the spatial pattern of perfusion response evoked by stimulation of a single eye to be similar to that seen during binocular stimulation, despite a substantial reduction in the metabolic response (compared to binocular stimulation) in columns projecting to the occluded eye. Since the BOLD signal reflects changes in the level of venous deoxygenated hemoglobin, this gratuitous hyperperfusion in unstimulated ocular dominance columns could be expected to result in a higher BOLD signal at a given level of perfusion increase (considering spatial averages over multiple columns, which would be applicable at commonly used spatial resolutions in fMRI).

The present study examines joint changes in perfusion and BOLD signals during monocular and binocular stimulation, to test the hypothesis that spatial decoupling of flow and metabolic responses during stimulation of only a partial subset of the columnar regions distributed within primary visual cortex leads to a significant shift in the ratio between spatially averaged BOLD and perfusion signals (measured using arterial spin-labeling). By combining quantitative MRI-based measures of these two physiological quantities, we hope to provide new insight into the spatial precision with which cerebral blood flow is regulated, as well as factors which determine BOLD contrast amplitude in cortical tissues exhibiting columnar organization.

## 2. METHODS

### 2.1. Subjects

Eight healthy subjects (five males and three females) 24 ± 2.6 years old, one left eye and hand dominant (male) and seven right eye and hand dominant, participated in the study. The subjects did not suffer from any known visual deficits except myopia (MRI-compatible corrective glasses were fitted in these cases). All gave informed consent and the project was approved by the Comité mixte d’éthique de la recherche du Regroupement Neuroimagerie/Québec. Data from two of the subjects was not analyzed due to the poor quality of the arterial spin-labeling (perfusion) data.

### 2.2. Visual stimulation

Subjects were fitted with a neoprene rubber mask which allowed occlusion of one eye by a removable patch. The patch was applied and removed as needed between the appropriate scans, by an experimenter, from the back of the scanner bore.

Each scanning session included eight six-minute acquisitions, during which alternating one-minute blocks of baseline (uniform grey screen with central fixation point) and one-minute blocks of visual stimulation (black and white checkerboard reversing contrast at a rate producing four white periods per second within a square) were presented, starting with baseline. During each scanning run, the subject received either binocular (B) or monocular (M) stimulation to their nondominant eye with separate scans conducted in the following order: B-B-M-M-B-B-M-M. Subjects were instructed to direct their gaze at the central fixation point throughout all scans. The nondominant eye was selected for monocular stimulation to maximize the difference in activation between the monocular and binocular trials given that there may presumably be more extensive activation of V1 for the dominant eye [10].

### 2.3. MRI data acquisition

MRI data acquisition was carried out using a Siemens Trio 3 Tesla MRI system, at software revision VA25A. Images reflecting relative perfusion were acquired using a PICORE/Q2TIPS arterial spin-labeling (ASL) acquisition [11, 12]. The spatial resolution was 3.4 mm × 3.4 mm on a 64 × 64 matrix, with 10 slices of 5 mm thickness. Other sequence parameters included TR/TE/alpha = 2 s/19 ms/90° and TI1/TI2 = 700 ms/1400 ms. A slab thickness of 200 mm was used, with a 10 mm gap between the top of the label slab and the most inferior image slice. The Q2TIPS stop time was 1350 ms. MR signals were received using an eight-channel receive-only head coil, with excitation and labeling performed using the system body coil.

A T1-weighted structural scan was also acquired for later use in spatial normalization. These scans were at 1 mm isotropic resolution, acquired using an MPRAGE sequence with TI/TR/TE/alpha = 900/2300/2.94/9. Voxel size was 1.0 × 1.0 × 1.2 mm.

### 2.4. Analysis

Flow and BOLD images were generated using the “surround subtraction” approach described in Wong et al. [13], reducing artifactual flow signals during periods of BOLD signal transition. The sequence of flow images was generated by computing the difference between each image and the average of the previous and subsequent images. The sequence of BOLD images was computed by adding each image to the average of its two neighbors. For each run, the effect and standard error maps were then generated by fitting a linear signal model to each voxel in the flow and BOLD series. The model consisted of a term representing the three task epochs in the run convolved with a dual gamma function including positive response plus undershoot [14], plus a third-order polynomial. Multiple runs for each subject were then combined using a mixed-effects model, followed by spatial normalization to the MNI 152 brain and combination of normalized maps for different subjects again using a mixed-effects model (as described in Worsley et al. [15]). Individual and multisubject maps were then thresholded at *P* = .001 significance with correction for multiple comparisons using the stat_threshold routine of the fMRIstat software package [15].

Regions of interest (ROIs) were generated using the NeuroLens software package (www.neurolens.org). Average BOLD statistical maps for each subject were used to make a V1 ROI by thresholding as described above. Voxels exceeding threshold in the BOLD map but located outside the banks of the calcarine sulcus as visualized in the T1-weighted structural scan were manually edited from the ROI, to ensure that the signals extracted were associated with primary visual cortex and therefore contained tissue organized as ocular dominance columns. The effect values were then averaged within the ROI for each functional scan and tabulated as effect size ± standard error. Values were converted to percent change as needed by dividing the effect size by the constant (DC) term fit during linear modeling and multiplying by 100.

## 3. RESULTS

**Figure 1:**
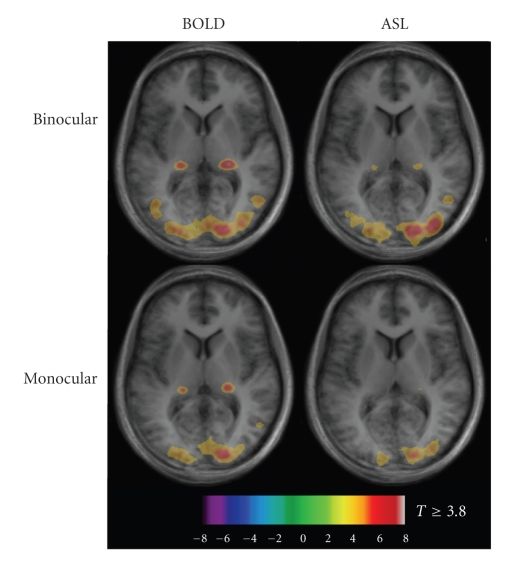
Mixed-effects response maps for BOLD and flow changes in response to monocular and binocular visual stimulation (n = 6). Spatial extent and intensity are greater for binocular stimulation than for monocular, for both BOLD and flow signals. Thresholded activation maps are overlaid on average T1 maps for the six subjects.

Figure 1 shows mixed-effects BOLD and flow maps over all subjects for monocular and binocular stimulation. Occlusion of input to one eye reduced the amount of activation detected in extrastriate areas. However, the significance levels observed within primary visual cortex appeared similar during both monocular and binocular stimulation. 

**Figure 2:**
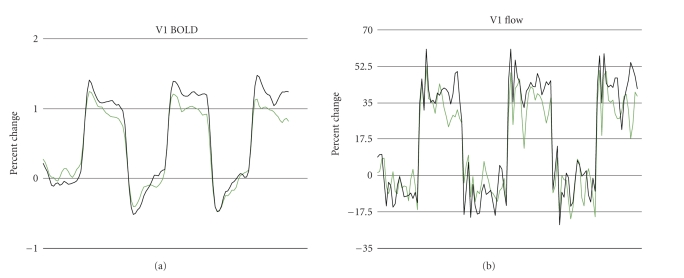
BOLD and flow signals (expressed as percent change; black = binocular, green = monocular) in response to monocular and binocular visual stimulation, averaged over all subjects (6 subjects × 4 averages per subject = 24 averages per signal). Initial overshoot and poststimulus undershoot are observed in BOLD signal for both monocular and binocular stimulation, as is typical for checkerboard stimulation.

Averaged time course signals for flow and BOLD are shown in Figure 2, revealing the initial BOLD signal overshoot and poststimulus undershoot commonly observed in visual cortex during checkerboard stimulation (seen here during both monocular and binocular stimulation). The flow signal illustrates the lower signal-to-noise ratio generally obtained in arterial spin-labeling measurements.

**Figure 3:**
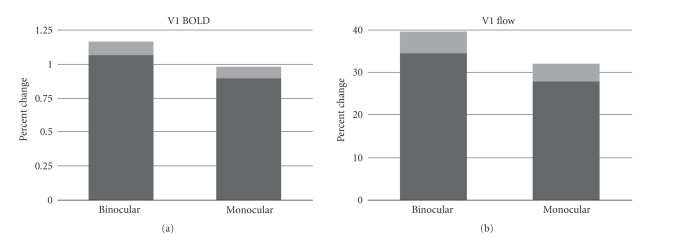
Percent change (±SE in lighter shade of gray) in BOLD and CBF signals in response to binocular and monocular stimulation. Responses evoked by binocular stimulation are significantly greater than those produced by monocular stimulation.

The bar graphs in Figure 3 show average percent changes in BOLD and flow signals within the V1 ROIs of all subjects. The average percent change in BOLD signal for monocular stimulation was 0.93±0.04, which was significantly (*P* < .05) less than the percent change of 1.11 ± 0.05 observed during binocular stimulation. The percent flow increase measured during monocular stimulation was 29 ± 2, also significantly less than the percent change of 37 ± 2 observed during binocular stimulation. Expressed as a percent reduction in the response amplitude, withholding input from one of the two eyes resulted in a 16 ± 6% decrease in the BOLD response and 19 ± 9% decrease in the perfusion response.

**Figure 4:**
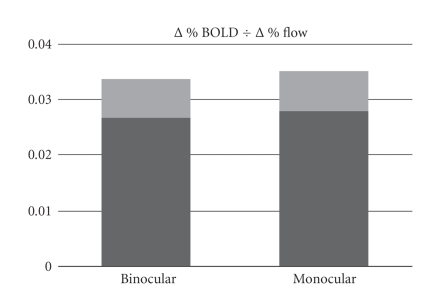
Percent change ratios (±SE in lighter shade of gray) for BOLD and CBF during binocular and monocular stimulation. There is no significant difference between the ratios for the two forms of stimulation, suggesting a comparable degree of flow-metabolism coupling throughout V1 regardless of the columnar fraction activated.

The percent changes in BOLD signal per percent signal increase in flow (i.e., the quotient Δ%BOLD ÷ Δ%flow) during monocular and binocular stimulation were found, respectively, to be 0.031 ± 0.004 and 0.030 ± 0.004 (Figure 4). The difference between these ratios was not statistically significant, failing to support any difference in flow-metabolism coupling during the two forms of stimulation.

## 4. DISCUSSION

In this study, we examined the coupling between BOLD and CBF responses in primary visual cortex during monocular and binocular stimulation. We found that the BOLD and CBF responses to monocular visual stimulation were both significantly reduced in V1 relative to the responses observed during binocular stimulation (Figure 3). The ratio of BOLD to CBF effect sizes did not differ significantly between the two stimulation conditions (Figure 4), indicating comparable coupling between flow and oxidative metabolism regardless of the columnar fraction that was activated.

The results obtained in the present study do not support the “strong” form of the theory that tissue perfusion is regulated only on a coarse spatial scale, irrespective of the spatial precision with which metabolism might change. This notion has been described previously as “watering the entire garden for the sake of one thirsty flower” [9]. To borrow the garden analogy, the experiments described here could be described as an attempt to measure the total water intake of the garden, as well as the runoff of unused water, to test this hypothesis. Our results are consistent with recent MRI studies showing that there is in fact sufficient spatial contrast in the perfusion response, as imaged using arterial spin-labeling, to resolve columnar structures in the visual cortex [6]. The study by Duong et al. [6] found that the early negative BOLD response (initial dip) also exhibited a high degree of spatial localization, whereas the late positive BOLD response was more diffuse. It is important to remember that the apparent resolution of each signal is dictated both by the underlying physiological regulatory precision and by the degree to which confounding vascular structures are superimposed on the pattern of parenchymal activation. Based on our results and those from Duong et al., it appears likely that the lack of precision in the late BOLD signal is due primarily to obscuring effects from the macrovascular anatomy, rather than a diffuse parenchymal BOLD effect. It has been suggested by other authors [16] that the increase in the apparent precision of the initial dip arises because BOLD signal increases in large draining veins do not arise until after the initial transit of blood through the local capillary bed postulated to take approximately one second.

Given that functional signals of interest may exhibit bias due to vascular anatomy, designating regions of interest using objective criteria is an important part of quantitative neuroimaging studies. In the present study, the use of phase-encoded retinotopic mapping to identify V1 in a separate mapping experiment would have been the optimal approach, since this procedure yields a set of voxels exhibiting a specific spatial trend in the polar angle or eccentricity of their projection in visual field that is unlikely to appear in a large vein. This would have led to excessively long scan sessions, however. Instead, we used the fact that the optic radiations terminate in the calcarine sulcus, making it very probable that activated regions lying in this anatomical zone are in fact part of primary visual cortex. It is still possible that the BOLD activation maps used to create ROIs based on a simple “activation minus baseline” contrast could contain a disproportionate contribution from large draining veins. These veins mix venous outflow from multiple visual areas, including regions which do not exhibit eye-specific columnar segregation, diluting any shift in flow-BOLD coupling present specifically in V1. In a pilot study of six subjects performed at 1.5T using retinotopic mapping to identify V1 (but performed using single-slice ASL at 1.5 T), we obtained a virtually identical result [17]. We therefore do not feel that the results of the present study are substantially impacted by our ROI selection procedure. Moreover, the relatively large voxel size and intense stimulus used in the present study yielded diffuse regions of robust activation that did not appear to be limited to macrovascular responses (as can occur at higher spatial resolutions or with weaker stimuli).

By measuring total flow and BOLD responses in V1 during activation of different columnar fractions, we were able to achieve high signal-to-noise ratios (SNRs) compared to studies that have used extremely high spatial resolution to actually resolve the columns. The purpose of the present study was to provide insight into two questions: the first is whether there is in fact a fundamental difference in the spatial precision with which perfusion and oxidative metabolism is measured; the second was to determine the impact of partial activation of a cortical columnar system on the BOLD signal characteristics observed at a customary fMRI spatial resolution. If there is indeed a profound mismatch in the spatial extent of increases in oxidative metabolism and flow, this should be apparent in the total average signal over V1. That none was found suggests that similar extents are likely to be found at higher spatial resolutions.

However, it is notable that while removal of input from one of the two eyes did result in a reduction of both BOLD and flow signals, the response decreased by much less than one half. This is consistent with detailed autoradiographic studies showing that pronounced ocular dominance segregation is mainly limited to cortical layer IV, with layers II and III actually exhibiting *higher* activation during binocular than monocular stimulation [10, 18]. This is consistent with later human neuroimaging studies, in which some regions showed higher apparent activity levels during the appropriate monocular stimulation than during binocular input [16, 19]. The columnar structure associated with ocular dominance is therefore most appropriately viewed as reflecting a moderate bias in overall selectivity, associated primarily with a single cortical sublayer, superimposed on numerous other modulating influences.

In light of the issues discussed above, it is clear that the columnar segregation of brain activation is not “all or nothing” during selective stimulation such as monocular or single-orientation conditions. Much of the early research in this area, performed using optical imaging methods capable of producing compelling maps of the columnar architecture, examined the perfusion of orientation domains (e.g., Malonek and Grinvald [9]) and not ocular dominance columns although a number of authors have imaged ocular dominance using a variety of other methods [5, 6, 10, 19, 20]. It would therefore be informative to replicate the present study using different combinations of oriented stimuli. It is also possible that certain stimuli might achieve a higher degree of selectivity than the ones used in this and prior studies. Perhaps under such conditions a small difference in flow-BOLD coupling might become detectable. Future investigation of this topic might include the use of different stimuli designed to selectively activate pathways involved in stereopsis.

## 5. CONCLUSION

Our results do not support the theory of spatially decoupled responses in blood flow and oxidative metabolism during activation of a subset of cortical ocular dominance columns. The limited impact of monocular blockade on flow and BOLD response amplitudes is also demonstrated, and should serve as a caution that ocular dominance contrast is likely to be faint in hemodynamic imaging methods.
